# Prevalence and Clinical Characteristics including Patterns of Antihypertensive Drug Administration of the Different Home Blood Pressure Phenotypes in Treated Hypertensive Patients

**DOI:** 10.1155/2022/6912839

**Published:** 2022-12-08

**Authors:** Chavalit Chotruangnapa, Tossaporn Thammarux, Piyawan Thongdang

**Affiliations:** Division of Hypertension, Department of Medicine, Faculty of Medicine Siriraj Hospital, Mahidol University, Bangkok, Thailand

## Abstract

Quality and quantity of home blood pressure (BP) control are important for optimizing hypertensive treatment. The prevalence and associated clinical characteristics of the different home blood pressure phenotypes in treated hypertensive patients were not elucidated. This study was conducted in Siriraj Hospital, Thailand from 2019 to 2020. We included treated hypertensive patients with ≥1 antihypertensive drug and had self-home BP measurement data. Both traditional (office BP < 140/90 mmHg and home BP < 130/80 mmHg) and new BP targets (office and home BP < 130/80 mmHg) were used for the classification of BP phenotypes. Home BP phenotypes consisted of controlled hypertension (all home BPs achieved home BP targets), isolated uncontrolled morning hypertension (MoHT) (only morning BP was above home BP targets), isolated uncontrolled evening hypertension (EHT) (only evening BP was above home BP targets), and combined morning-evening uncontrolled hypertension (MoEHT) (all home BPs were above home BP targets). Our study included 1,406 patients. The total mean age was 62.94 ± 13.97 years. There were 39.40% men. The prevalence of each home BP phenotype (by traditional BP target) was 55.76%, 12.66%, 7.40%, and 24.18% in controlled (home) hypertension, MoHT, EHT, and MoEHT, respectively. Classical BP control status was 35.21% well-controlled hypertension, 30.01% white-coat uncontrolled hypertension, 9.74% masked uncontrolled hypertension, and 25.04% sustained uncontrolled hypertension. The multivariable analysis showed the significantly associated factor of MoHT was the presence of previous cardiovascular disease (adjusted OR 5.54, 95% CI (2.02–15.22); *p* value = 0.001). Taking once-daily long-acting antihypertensive drugs in the morning were significantly associated with both EHT (adjusted OR 0.20, 95% CI (0.05–0.82); *p* value = 0.025) and MoEHT (adjusted OR 0.20, 95% CI (0.04–1.00); *p* value = 0.049). These results were consistent in groups classified by new home BP target <130/80 mmHg.

## 1. Introduction

Nowadays home blood pressure monitoring (HBPM) plays a role in the diagnosis and management of hypertension because home blood pressure (BP) is strongly associated with cardiovascular outcomes [1]. HBPM does not only improve hypertension awareness and adherence that result in better BP control [2, 3] but it also classifies white-coat effect/white-coat hypertension and masked hypertension in addition to office BP measurement. Several current guidelines recommend using home BP as the target BP for the treatment of hypertension in order to improve the quality of BP control in hypertensive patients [4–7]. Previous studies demonstrated a positive association between morning BP or morning hypertension at home and cardiovascular disease, stroke [8, 9], and chronic kidney disease [10]. The Ohasama Study showed the predictive power of both morning and evening hypertension which were assessed by HBPM for stroke in hypertensive patients [11]. Patients with morning or evening or a combination of morning and evening hypertension might have different clinical characteristics. Some studies revealed that regular alcohol drinking [12], antihypertensive drug regimens, diabetes mellitus, male gender, and kidney disease affected home BP phenotypes [13]. Since the growing pieces of evidence have supported intensive BP control for the prevention of cardiovascular morbidities and mortality [14]. Some recent guidelines suggest reducing office and home BP to <130/80 mmHg [4, 15], which is the new BP target. According to the difference between the traditional BP target (office BP target is <140/90 mmHg and home BP target is <135/85 mmHg) and the new BP target (both office and home BP targets are <130/80 mmHg), this study aimed to evaluate the prevalence of BP control status including different home BP phenotypes and to investigate whether clinical characteristics and treatment associated with the different home BP phenotypes in treated hypertensive patients.

## 2. Materials and Methods

### 2.1. Study Population

A cross-sectional study was conducted at Siriraj Hospital, Mahidol University, Thailand. The medical records of the hypertensive patients who were treated at the hypertension clinic from 1 January 2018 to 31 December 2020 were retrospectively reviewed. The eligible participants were the patients who met all inclusion criteria: (1) age of at least 18 years, (2) diagnosed with hypertension and treated with at least one antihypertensive drug for at least 4 weeks, and (3) completed data of home BP in morning and evening times for at least 3 consecutive days and at least 12 home BP records (≥6 home BP records in each morning and evening) [16]. Patients with pregnancy, end-stage kidney disease, secondary hypertension, and unable measurement of BP at the brachial artery were excluded. Our study was approved by the Siriraj Institutional Review Board (SIRB) (COA no. Si 356/2022).

### 2.2. Blood Pressure Measurement

The physicians and well-trained nurses regularly generally informed hypertensive patients in the hypertension clinic on the standard method of HBPM and asked them to measure and record their home BPs twice, 1 minute apart each morning and evening times for 3 to 7 consecutive days before the next appointed visit. BP measurement was performed after at least 5 minutes of resting period in a sitting position on the chair with back support. The appropriate-sized cuff was placed on their arms at the same level as the heart. Because all participants had antihypertensive drugs at different times, hence the participants were informed to measure their morning BP before taking antihypertensive drugs and before bedtime which was defined as evening BP. If some antihypertensive drugs were administrated before bedtime, evening BP was suggested to measure before taking these drugs. The validated oscillometric BP devices for HBPM were Omron HEM-7130 and HEM 7211 (Omron Healthcare Co. Ltd.) because we practically provided the home BP devices to most patients in the hypertension clinic. The results of the home BPs of the patients had been recorded in the medical records of Siriraj hospital. The average home morning and evening BPs were calculated from all numbers of BPs each time. Average daytime home BP was calculated from the average of mean home morning and evening BP.

Office BP measurement followed the standard technique of accurate attended BP measurement as per international guideline's recommendation on the visit day. The validated oscillometric BP devices (Omron HBP-300 and HBP-110; Omron Healthcare Co. Ltd.) were used for BP measurement in a sitting position after at least 3 to 5 minutes of rest. The last two office BPs were calculated as average office BP.

### 2.3. Definition of Different Home Blood Pressure Phenotypes According to Home Blood Pressure Control

We classified home BP phenotypes depending on morning and evening BP control into four following phenotypes: (1) controlled hypertension (all average morning and evening SBPs were <135 mmHg and DBPs were <85 mmHg); (2) isolated uncontrolled morning hypertension (MoHT) (Only average morning SBP was ≥135 mmHg and/or DBP was ≥85 mmHg but average evening SBP was <135 mmHg and DBP was <85 mmHg); (3) isolated uncontrolled evening hypertension (EHT) (Only average evening SBP was ≥135 mmHg and/or DBP was ≥85 mmHg but average morning SBP was <135 mmHg and DBP was <85 mmHg); and (4) combined morning-evening uncontrolled hypertension (MoEHT) (all average morning and evening SBPs were ≥135 mmHg and/or DBPs were ≥85 mmHg).

In addition, we also used the new recommended BP target of the 2017 American College of Cardiology (ACC)/American Heart Association (AHA) hypertension guideline [4] for classifying the following 4 home BP phenotypes: (1) controlled hypertension (all average morning and evening SBPs were <130 mmHg and DBPs were <80 mmHg); (2) isolated uncontrolled morning hypertension (MoHT) (Only average morning SBP was ≥130 mmHg and/or DBP was ≥80 mmHg but average evening SBP was <130 mmHg and DBP was <80 mmHg); (3) isolated uncontrolled evening hypertension (EHT) (Only average evening SBP was ≥130 mmHg and/or DBP was ≥80 mmHg but average morning SBP was <130 mmHg and DBP was <80 mmHg); and (4) combined morning-evening uncontrolled hypertension (MoEHT) (all average morning and evening SBPs were ≥130 mmHg and/or DBPs were ≥80 mmHg).

### 2.4. Definition of Different Classical Blood Pressure Phenotypes According to Both Office and Home Blood Pressure Control

Four classical BP phenotypes, which were well-controlled hypertension, white-coat uncontrolled hypertension (WCHT), masked uncontrolled hypertension (MHT), and sustained uncontrolled hypertension (SHT) were classified according to traditional and new BP targets. Traditionally, the office BP target was <140/90 mmHg, and the home BP target was <135/85 mmHg. The new BP target was <130/80 mmHg of both office and home BPs. Thus, well-controlled hypertension was defined as all office and average daytime home BPs achieved the BP targets. WCHT was the condition that had abnormally higher office BP than targeted office BPs while average daytime home BP still achieved the home BP targets. MHT was defined as office BP being within the office BP targets but home BP being above the home BP targets. The definition of SHT was the condition that had both higher office and home BPs than the BP targets.

### 2.5. Antihypertensive Drugs Regimen

To evaluate the pattern of prescribed antihypertensive drugs in routine clinical practice and the association between antihypertensive drug regimens and home BP phenotypes, we classified antihypertensive drugs into two groups according to their pharmacokinetics (eg. duration of action). Long-acting antihypertensive drugs were defined as their duration of BP reduction was 24 hours or more. Short-acting antihypertensive drugs were defined as their duration of action was less than 24 hours. Furthermore, the regimen of medical treatment was divided into the following 3 groups: (1) only morning drug administration which was defined as taking a once-daily drug before noon (ante meridiem); (2) only evening drug administration which was defined as taking once-daily drug after noon (post meridiem); and (3) both morning and evening drug administration.

Because of the effect of antihypertensive drugs' doses on BP values, trough-to-peak ratio, and blood pressure variability [17, 18], we used the two steps for the calculation of the proportion of antihypertensive doses in each morning and evening times to 24 hours. In the first step, the total antihypertensive therapeutic intensity score (total TIS) was calculated in the individuals. The formula of total TIS [19] was shown in the following equation:

(1) $$\begin{array}{c} total\;antihypertensive\;therapeutic\;intensity\;score=\sum \frac{Actual\;da\;ily\;do\;se}{Recommende\;d\;maximal\;da\;ily\;do\;se}\text{.} \end{array}$$

Second step, the TIS of each morning and evening drug administration was divided by total TIS.

### 2.6. Data Collection

The information of all participants was collected from the medical records of Siriraj hospital. Baseline information consisted of age, gender, comorbidities including the previous history of cardiovascular disease, history of smoking and alcohol drinking, body weight, height, office, and home BPs, and detail of antihypertensive drug prescription was collected. Cardiovascular disease defines as the presence of at least one of the following diseases: myocardial infarction, heart failure, ischemic and hemorrhagic stroke. The mineralocorticoid receptor antagonist was only spironolactone due to its availability in Thailand. The other class of antihypertensive drugs included centrally acting alpha-II agonists and direct vasodilators.

### 2.7. Statistical Analysis

The previous study reported that the proportion of BP control rate by achieving targeted BP at home was 57% [20]. We expected that the prevalence of well-controlled hypertension was 5% less than the study's prevalence so the expected prevalence was 52%. The estimated sample sizes of at least 1,038 participants were required to detect the difference of 5% with 80% power using a 5%-level two-sided test.

Descriptive statistics were used for the analysis of baseline characteristics data. Continuous variables, such as age and body mass index, were expressed as mean ± standard deviation or median (interquartile range) depending on the data's distribution. Categorical variables were presented as numbers and percentages. One-way ANOVA (analysis of variance) and the Kruskal–Wallis test were used to compare the continuous data between all groups according to normal or non-normal distribution, respectively. Because multiple analyses were performed, the statistically significant level was adjusted by Bonferroni correction. The Chi-square test was analyzed for the comparison of categorical variables. The association between clinical characteristics including treatment and home BP phenotypes was analyzed by the univariable and multivariable multinomial logistic regression model. Because of using the two different thresholds of home BP targets (<140/90 and <130/80 mmHg) for the classification of home BP phenotypes, the results were separately analyzed and presented. Statistical analyses were performed by Stata Statistical Software Version 17. (StataCorp LLC, College Station, TX). A *p* value of less than 0.05 was considered statistically significant.

## 3. Results

### 3.1. Baseline Characteristics and Home BP Phenotypes

After screening 1,606 hypertensive patients with had completed home BP data, we excluded 167 hypertensive ones without pharmacologic treatment and 33 ones with incomplete home BP data. Thus, this study included 1,406 treated hypertensive patients. The mean age of all groups was 62.94 ± 13.97 years. 554 (39.40%) men and 852 (60.60%) women were enrolled. The mean body mass index (BMI) of all groups was 25.54 ± 4.52 kg/m^2^. The patients with abnormally high BMI or overweight (BMI ≥ 23 kg/m^2^) were 69.65%. Dyslipidemia (79.71%) was the most comorbidity and 23.16% of all patients had diabetes mellitus. A previous history of cardiovascular disease was found in 9.41% of all patients and the proportion of patients with chronic kidney disease (CKD) was 17.39%. The mean number of antihypertensive drug classes was 2.13 ± 1.09 classes for total patients. Dihydropyridine calcium channel blocker (DHP-CCB) and angiotensin-II receptor blocker (ARB) were mainly used for the treatment of hypertension. The average office BP was 142.25 ± 15.89/78.67 ± 11.94 mmHg and the average daytime home BP was 128.05 ± 11.65/76.10 ± 10.11 mmHg. The detail of overall baseline characteristics was shown in Table 1.

Four home BP phenotypes were classified by home BP target of <135/85 mmHg. The different characteristics of these groups was shown in Table 1. The proportion of controlled hypertension, MoHT, EHT, and MoEHT was 55.76%, 12.66%, 7.40%, and 24.18%, respectively. Patients with MoEHT had the youngest age (60.47 ± 15.16 years). The proportion of overweight was significantly higher in MoHT (75%) and MoEHT (74.39%) groups than controlled hypertension group. Chronic kidney disease was more common in patients with EHT (26.92%) and MoEHT (22.35%). Alcoholic beverage was drunk in the highest proportion in the MoEHT group. Both patients with EHT (2.36 ± 1.17) and MoEHT (2.28 ± 1.17) took the higher numbers of antihypertensive drug classes compared to ones with controlled hypertension. Mineralocorticoid receptor antagonist (MRA) was significantly used in EHT and MoEHT groups, moreover, peripheral alpha-1 blockers were more frequently used in MoHT, EHT, and MoEHT groups. Both EHT and MoEHT groups had a significantly higher ratio of presence of albuminuria than the controlled hypertensive group but the only MoEHT group more commonly found albuminuria than the MoHT group. There were significantly different office BPs between the four home BP phenotypes. In addition, Average daytime BPs significantly increased from controlled hypertension, MoHT, and EHT to MoEHT groups. When reclassifying home BP phenotypes by using a new threshold of a home BP target of <130/80 mmHg, the result was similar to the aforementioned result using a home BP target of <135/85 mmHg. Supplementary Table 1 revealed the baseline characteristics of all reclassified groups.

### 3.2. Antihypertensive Drugs Regimens in Different Home BP Phenotypes

The overall results revealed that most treated hypertensive patients took once-daily antihypertensive drugs (57.61%). The most common timing antihypertensive drug administration was morning (42.75%). 57.25% of all patients had at least 1 antihypertensive drug taken in the evening. To determine the ratio of the evening dose of prescribed antihypertensive drugs to the total daily dose of all prescribed antihypertensive drugs, we calculated by the following formula: TIS of all antihypertensive drugs taken in the evening was divided by TIS of total daily doses of all antihypertensive drugs. The overall result showed that the evening dose of antihypertensive drugs was 33.52% of the daily dose of all prescribed antihypertensive drugs. Most prescribed antihypertensive drugs were long-acting.  Table 2 showed the results in all home BP phenotypes according to the home BP target of <135/85 mmHg. MoHT, EHT, and MoEHT groups had a significantly higher proportion of evening drug administration than the controlled hypertensive group. A higher dose of evening drug administration was significantly used in MoHT and MoEHT groups than in the controlled hypertensive group. Supplementary Table 2 showed the results in all groups that were classified by home BP target of <130/80 mmHg. Most results were similar to the results in Table 2. After group reclassification had been done by the new threshold of home BP target, only EHT and MoEHT groups had a significantly higher proportion of evening antihypertensive drug administration as well as evening doses than in the controlled hypertensive group.

### 3.3. Prevalence of Classical Blood Pressure Phenotypes According to Both Office and Home Blood Pressure Control

According to the office BP target of <140/90 mmHg and home BP target of <135/85 mmHg, there was 35.21% of well-controlled hypertension, 30.1% of WCHT, 9.74% of MHT, and 25.04 of SHT (Table 3). The proportion of these four groups was changed when using the new threshold of office and home BP target of <130/80 mmHg. The prevalence of well-controlled hypertension, WCHT, MHT, and SHT was 12.52%, 32.86%, 5.90%, and 48.72%, respectively (Supplementary Table 3).

Using traditional BP targets (office BP target of <140/90 mmHg and home BP target of <135/85 mmHg (Table 4), the patients with controlled both morning and evening home BPs were mostly found in well-controlled hypertension (88.26%) and WCHT groups (82.23%) in spite of the fact that they were not found in MHT and SHT groups. There was a gradually increased proportion of patients with MoHT from the group of well-controlled hypertension (7.27%), WCHT (9.95%), and MHT (17.52%) to SHT (21.59%). The proportion of EHT was 4.44% in the group with well-controlled hypertension, 7.82% in WCHT, 13.14% in MHT, and 8.81% in SHT groups. MoEHT was only found in MHT (69.34%) and SHT (69.6%) groups. Supplementary Table 4 showed that the results by using a new BP target of <130/80 mmHg were the same as mentioned above.

### 3.4. Association of Clinical Factors and Home BP Phenotypes

The results of univariable and multivariable regression for determining the association of clinical factors and home BP phenotypes which were classified by home BP target of <135/85 mmHg were shown in Tables 5 and 6, respectively. After office BP and average daytime home BP were adjusted in the multivariable regression model, only the presence of previous cardiovascular disease was significantly associated with MoHT (adjusted OR 5.54, 95% CI (2.02–15.22); *p* value = 0.001). Taking once daily long-acting antihypertensive drugs in the morning had a significant inverse association with both EHT (adjusted OR 0.20, 95% CI (0.05–0.82); *p* value = 0.025) and MoEHT (adjusted OR 0.20, 95% CI (0.04–1.00); *p* value = 0.049). These results were consistent in spite of reclassifying groups depending on the new home BP target of <130/80 mmHg (Supplementary Tables 5 and 6).

## 4. Discussion

Several present guidelines recommend lowering office and out-of-office BPs to each target BPs [4–7] due to out-of-office BP better predicting future cardiovascular events than office BP [1]. This study showed the prevalence of BP control by using the different BP targets which were the traditional BP target (office BP of <140/90 mmHg and home BP of <130/80 mmHg) and the new BP target (both office and home BPS of <130/80 mmHg). This study did not only classify all treated hypertensive patients into different four BP phenotypes by using average office BP and average daytime home BP but the average morning BP and evening BP were used for allocating these patients into different home BP phenotypes. The prevalence of well-controlled hypertension defined by <140/90 mmHg in average office BP and <135/85 mmHg in average daytime home BP was 35.21%. This finding was discordant with the study of Montrivade et al. which showed that the proportion of well-controlled hypertension was 30% [21] and the prevalence of the other classical BP phenotypes (WCHT, MHT, and SHT) was also quite similar. The changing prevalence of each classical BP phenotype defined by the new and more intensive office and home BP target of <130/80 mmHg was presented in this study. The rate of well-controlled hypertension greatly decreased from 35.21% to 12.52% and the rate of SHT increased from 25.04% to 48.72%. The different prevalence of reclassification of classical BP phenotypes especially in Thai-treated hypertensive patients was supported by Buranakitjaroen et al. [20] which demonstrated the change of prevalence of BP control when using different BP thresholds.

BP variability is suggested to be taken into account in the treatment of hypertension because there have been supporting pieces of evidence of the cardiovascular prognostic power of BP variability including diurnal BP change [22–24]. In addition, The Ohasama Study revealed the effect of morning and evening home BP on stroke [11]. It could be implied that uncontrolled home BP in either morning or evening was related to incident stroke and most home BPs should be achieved the goal BP. Considering the particular uncontrolled hypertension, we were able to divide it into 3 phenotypes in our study. They consisted of MoHT, EHT as well as MoEHT. MoEHT had the highest proportion among them. The result also revealed the proportion of different home BP phenotypes in each classical BP phenotype. Our study showed the presence of MoHT and EHT in well-controlled hypertension and WCHT groups although achieving a home BP target is one criterion for well-controlled hypertension and WCHT. Using average daytime BP to represent home BP in this study is the reason that explains this finding. Controlled (home) hypertension was commonly found in well-controlled hypertension and WCHT groups. On the contrary, MoEHT was commonly found in MHT and SHT groups.

There were several different characteristics between the 4 groups. Patients with MoEHT had the youngest age. Body mass index gradually increased from controlled hypertension, MoHT, EHT, to MoEHT. Some previous studies supported that overweight and obesity were strongly associated with poor BP control [25, 26]. Chronic kidney disease was found in a higher proportion in uncontrolled hypertensive groups, especially EHT and MoEHT. Chronic kidney disease was significantly associated with uncontrolled BP [27]. Masked uncontrolled hypertension was also common in patients with chronic kidney disease and reduced glomerular filtration rate [27, 28]. The reason why the EHT had the highest proportion of chronic kidney disease remained unclear because most previous studies evaluated diurnal BP variation by 24-hour ambulatory BP monitoring and reported average daytime BP, average nighttime BP, and average 24-hour BP [29, 30] and some studies investigated BP control rate by using mean overall home BP [31, 32]. Guidelines for hypertension treatment recommend limiting alcohol intake because of its effect on BP control [4–7]. The study showed that alcohol drinking was more commonly found in MoEHT than in the other groups. The average office BP and average daytime BP in the MoEHT group were the highest among the four groups despite the fact that the MoEHT group had a higher number of antihypertensive drug classes and a higher proportion of taking mineralocorticoid receptor antagonist and peripheral alpha-I receptor blockers, which were not the main class of antihypertensive drugs and were used for add-on therapy. The finding demonstrated the inadequate hypertensive treatment of the patients in sustained uncontrolled hypertensive groups. The overall mean number of antihypertensive drug classes was 2.13 ± 1.09. It was concordant with the recommended combination therapy of antihypertensive drugs of present guidelines [5–7]. Uncontrolled hypertensive groups had a higher proportion of evening antihypertensive drug administration than controlled hypertensive ones. The evening to total daily dose ratio of antihypertensive drugs in MoHT and MoEHT was significantly higher than controlled hypertension. The long-acting antihypertensive drug was prescribed in higher proportion than the short-acting drug. It indicated that the prescribed antihypertensive drug regimen for the treatment of hypertension in routine practice followed the recommendation of recent guidelines [4–7]. Albuminuria is a surrogate marker of kidney damage and cardiovascular disease [33]. It was significantly presented in EHT and MoEHT compared to controlled hypertension because these two groups had a higher proportion of chronic kidney disease than the others. All aforementioned findings indicated uncontrolled hypertensive groups, in particular MoEHT, had more severity of hypertension than ones with controlled BP.

In addition, the analysis for investigation of the association of clinical factors and home BP phenotypes was performed by comparing each uncontrolled hypertensive group with the controlled hypertensive group. On the basis of multivariable multinomial logistic regression analysis with adjustment of average office and daytime home BPs, we separately discussed the association in each home's BP phenotypes. In the MoHT group, the multivariable analysis showed that a history of previous cardiovascular disease was the significantly independent associated factor. Even though the previous evidence supported that morning hypertension was strongly related to stroke [9, 34, 35], It could not show the causal effect of cardiovascular disease and MoHT in this study because of the limitation of the cross-sectional design. For EHT and MoEHT groups, the independently associated clinical factor was taking at least 1 long-acting antihypertensive drug once daily in the morning. Antihypertensive drugs were an important factor in home BP control since previous studies of chronotherapy of hypertension indicated that evening or bedtime administration of antihypertensive drugs improved morning BP control [36–38]. Because of the long duration of action of antihypertensive drugs that were taken in the morning, the evening home BP was controlled to achieve the BP target. This reason may explain the finding of the inverse association between this factor and EHT. Furthermore, the clinical factor remained inversely associated with MoEHT. Patients with MoEHT had more severe hypertension and more numbers of comorbidities so a more complex drug regimen and polypharmacy might be necessary to control their diseases. Our results showed the gradually increased proportion of taking at least 1 long-acting antihypertensive drug in both morning and evening from controlled hypertension, MoHT, EHT to MoEHT while once-daily therapy in the morning of at least 1 long-acting antihypertensive drug had the lowest proportion of prescription in the MoEHT group. The reverse causality of the factor which is taking at least 1 long-acting antihypertensive drug once daily in the morning and MoEHT probably explained this inverse association between them.

The strength of this study was the additional information on home BP control by consideration of the component of BP at each time. The study also emphasized the different phenotypes of uncontrolled hypertension. Although EHT was a minor population in uncontrolled hypertensive groups similar to the Ohasama study [11] and had been usually ignored, this condition was not benign. This study also analyzed the effect of pattern antihypertensive drug administration and pharmacokinetics which might be an important factor for circadian BP change and control. However, the study had several limitations. First, this study design was cross-sectional. It limited the interpretation of the association of results and could not directly identify the causal relationship between factors and outcomes. Second, the definition of evening antihypertensive drug administration included the variation of time to take medication after noon while evening BP was defined as measured BP before bedtime. Thus, evening BP might be affected by the peak effect of taking antihypertensive drugs after dinner in some patients. Finally, there were some effects of unmeasured confounding factors, for example, drug adherence, nonpharmacologic intervention, and daily activities, which might affect the association of clinical factors and home BP phenotypes.

In conclusion, the rate of well-controlled BP (both average office and daytime home BPs achieved office and home BP targets) remained low in treated hypertensive patients. EHT phenotype had the lowest proportion among uncontrolled hypertensive groups. There were several different clinical characteristics in different three phenotypes of uncontrolled home BPs. The independent clinical factors associated with MHT were a previous history of cardiovascular disease. Taking at least 1 long-acting antihypertensive drug in the morning is significantly associated with EHT and MoEHT. However, further well-designed studies for investigating the effect of chronotherapy and pharmacokinetic properties of antihypertensive drugs on home blood pressure control and long-term cardiovascular outcome in each home BP phenotypes are required.

## Figures and Tables

**Table 1:** Baseline characteristics of home blood pressure phenotypes by using a home blood pressure target of <135/85 mmHg.

Parameters	Home blood pressure phenotypes				Total	*p* value^*∗*^
	Controlled hypertension	Isolated uncontrolled morning hypertension	Isolated uncontrolled evening hypertension	Combined morning-evening uncontrolled hypertension		
Number (%)	784 (55.76)	178 (12.66)	104 (7.40)	340 (24.18)	1,406 (100)	—
Age (years)	63.73 ± 12.98	63.93 ± 14.77	63.38 ± 14.95	60.47 ± 15.16^†‡^	62.94 ± 13.97	0.006
Male *N* (%)	290 (36.99)	77 (43.26)	43 (41.35)	144 (42.35)	554 (39.40)	0.219
BMI (kg/m^2^)	25.13 ± 4.36	25.44 ± 3.69	25.78 ± 4.38	26.48 ± 5.16^†^	25.54 ± 4.52	0.001

*Comorbidities N (%)*						
Overweight (BMI ≥ 23 kg/m^2^)	508 (66.41)	129 (75.00)^†^	69 (69.70)	244 (74.39)^†^	950 (69.65)	0.022
Diabetes mellitus	173 (22.09)	41 (23.30)	29 (27.88)	82 (24.12)	325 (23.16)	0.576
Dyslipidemia	600 (76.53)	139 (78.98)	85 (81.73)	271 (79.71)	1,095 (77.99)	0.475
Previous CVD^*∗∗*^	67 (8.56)	22 (12.50)	9 (8.65)	34 (10.00)	132 (9.41)	0.416
Chronic kidney disease	112 (14.30)	28 (15.91)	28 (26.92)^†‡^	76 (22.35)^†^	244 (17.39)	<0.001
Obstructive sleep apnea	45 (5.77)	13 (7.39)	9 (8.65)	34 (10.00)	101 (7.21)	0.082
Current smoking *N* (%)	13 (1.75)	0 (0)	1 (0.99)	9 (2.79)	23 (1.73)	0.144
Alcohol drinking *N* (%)	24 (3.24)	6 (3.57)	3 (2.97)	24 (7.43)^†^	57 (4.28)	0.015
Numbers of antihypertensive classes	2.02 ± 1.00	2.19 ± 1.21	2.36 ± 1.17^†^	2.28 ± 1.17^†^	2.13 ± 1.09	0.002
Diuretics *N* (%)	81 (10.33)	25 (14.04)	19 (18.27)	45 (13.24)	170 (12.09)	0.070
MRA *N* (%)	20 (2.55)	5 (2.81)	8 (7.69)^†^	21 (6.18)^†^	54 (3.84)	0.004
ACEIs *N* (%)	118 (15.05)	25 (14.04)	13 (12.50)	54 (15.88)	210 (14.94)	0.839
ARBs *N* (%)	380 (48.47)	87 (48.88)	57 (54.81)	158 (46.47)	682 (48.51)	0.527
DHP-CCBs *N* (%)	571 (72.83)	131 (73.60)	81 (77.88)	263 (77.35)	1,046 (74.40)	0.349
Non-DHP-CCBs *N* (%)	46 (5.87)	10 (5.62)	6 (5.77)	25 (7.35)	87 (6.19)	0.786
BBs *N* (%)	233 (29.72)	57 (32.02)	27 (25.96)	94 (27.65)	411 (29.23)	0.635
Peripheral alpha-I blockers *N* (%)	96 (12.24)	36 (20.22)^†^	27 (25.96)^†^	89 (26.18)^†^	248 (17.64)	<0.001
Centrally acting alpha-II agonists *N* (%)	17 (2.17)	3 (1.69)	4 (3.85)	12 (3.53)	36 (2.56)	0.392
Direct vasodilators *N* (%)	18 (2.30)	10 (5.62)	3 (2.88)	15 (4.41)	46 (3.27)	0.075

*Laboratory result*						
FBS (mmol/l)	5.99 ± 1.19	6.05 ± 1.22	6.04 ± 1.34	6.05 ± 1.51	6.02 ± 1.29	0.787
HbA1C (%)	6.01 ± 0.78	6.08 ± 0.83	6.16 ± 0.88	6.07 ± 1.00	6.05 ± 0.85	0.148
Cholesterol (mmol/l)	4.71 ± 0.93	4.88 ± 1.10	4.67 ± 0.97	4.89 ± 1.11	4.77 ± 1.00	0.069
Triglyceride (mmol/l)	1.28 ± 0.59	1.42 ± 0.69	1.51 ± 0.72^†^	1.49 ± 0.78^†^	1.37 ± 0.67	<0.001
HDL-C (mmol/l)	1.54 ± 0.45	1.49 ± 0.40	1.42 ± 0.38	1.46 ± 0.52^†^	1.51 ± 0.46	<0.001
LDL-C (mmol/l)	2.62 ± 0.84	2.77 ± 0.99	2.61 ± 0.83	2.77 ± 0.91	2.68 ± 0.88	0.050
eGFR (ml/min/1.73 m^2^)	80.46 ± 21.29	77.49 ± 22.43	75.84 ± 24.88	76.68 ± 27.48	78.82 ± 23.40	0.077
Presence of albuminuria *N* (%)	132 (21.19)	31 (23.85)	26 (31.33)^†^	107 (37.41)^†‡^	296 (26.38)	<0.001

*Office BP*						
Average office SBP (mmHg)	138.89 ± 15.44	145.27 ± 13.87^†^	143.96 ± 14.98^†^	147.89 ± 16.23^†^	142.25 ± 15.89	<0.001
Average office DBP (mmHg)	76.20 ± 10.71	80.99 ± 12.55^†^	79.41 ± 12.18	82.93 ± 12.79^†^^^⁋^^	78.67 ± 11.94	<0.001

*Morning home BP*						
Average morning home SBP (mmHg)	121.55 ± 8.03	137.09 ± 8.06	127.56 ± 6.39	141.35 ± 11.29	128.75 ± 12.41	<0.001
Average morning home DBP (mmHg)	72.68 ± 7.72	82.66 ± 9.28	74.76 ± 8.08	85.30 ± 10.52	77.15 ± 10.35	<0.001

*Evening home BP*						
Average evening home SBP (mmHg)	120.36 ± 8.25	126.83 ± 7.08	137.11 ± 7.48	140.77 ± 10.70	127.35 ± 12.40	<0.001
Average evening home DBP (mmHg)	70.56 ± 7.79	75.02 ± 9.08	78.76 ± 9.52	84.27 ± 11.93	75.04 ± 10.86	<0.001

*Daytime home BP*						
Average daytime home SBP (mmHg)	120.95 ± 7.40	131.96 ± 6.15^†^	132.33 ± 6.02^†^	140.04 ± 10.28^†‡^^^⁋^^	128.05 ± 11.65	<0.001
Average daytime home DBP (mmHg)	71.62 ± 7.46	78.84 ± 8.62^†^	76.76 ± 8.28^†^	84.79 ± 10.44^†‡^^^⁋^^	76.10 ± 10.11	<0.001

BMI, body mass index; kg/m^2^, kilogram/meter^2^; CVD, cardiovascular disease; MRA, mineralocorticoid receptor antagonist; ACEIs, angiotensin-converting enzyme inhibitors; ARBs, angiotensin-II receptor blockers; DHP-CCBs, dihydropyridine calcium channel blockers; non-DHP-CCBs, nondihydropyridine calcium channel blockers; BBs, beta-blockers; FBS, fasting blood sugar; HbA1C, hemoglobin A1C; HDL-C, high-density lipoprotein-cholesterol; LDL-C, low-density lipoprotein-cholesterol; eGFR, estimated glomerular filtration rate; mg/g, a milligram of albuminuria/gram of urine creatinine; BP, blood pressure; SBP, systolic blood pressure; DBP, diastolic blood pressure; mmHg, millimeter of mercury. ^*∗*^*p* value for comparing parameters of all home blood pressure phenotypes. ^*∗∗*^included a previous history of myocardial infarction, heart failure, ischemic stroke, or hemorrhagic stroke. ^†^*p* value <0.05 for comparing to controlled hypertension. ^‡^*p* value <0.05 for comparing to isolated uncontrolled morning hypertension. ^^⁋^^*p* value <0.05 for comparing to isolated uncontrolled evening hypertension.

**Table 2:** The regimen of prescribed antihypertensive drugs of home blood pressure phenotypes by using a home blood pressure target of <135/85 mmHg.

Parameters	Home blood pressure phenotypes				Total	*p* value^*∗*^
	Controlled hypertension	Isolated uncontrolled morning hypertension	Isolated uncontrolled evening hypertension	Combined morning-evening uncontrolled hypertension		
Number (%)	784 (55.76)	178 (12.66)	104 (7.40)	340 (24.18)	1,406 (100)	—
Frequency of drug administration (times per day) *N* (%)						<0.001
1	505 (64.41)	91 (51.12)	51 (49.04)	163 (47.94)	810 (57.61)	
2	240 (30.61)	71 (39.89)	41 (39.42)	137 (40.29)	489 (34.78)	
3	33 (4.21)	9 (5.06)	8 (7.69)	25 (7.35)	75 (5.33)	
4	6 (0.77)	7 (3.93)	4 (3.85)	15 (4.41)	32 (2.28)	
Timing of antihypertensive drug administration *N* (%)						<0.001
Only morning administration	405 (51.66)	59 (33.15)	39 (37.50)	98 (28.82)	601 (42.75)	
Only evening administration	103 (13.14)	32 (17.98)	13 (12.50)	65 (19.12)	213 (15.15)	
Both morning and evening administration	276 (35.20)	87 (48.88)	52 (50.00)	177 (52.06)	592 (42.11)	
Evening drug administration *N* (%)	379 (48.34)	119 (66.85)^†^	65 (62.50)^†^	242 (71.18)^†^	805 (57.25)	<0.001
Proportion of TIS (evening) to TIS (24 hours)^‡^ (%)	27.44 ± 35.18	40.84 ± 36.34^†^	34.69 ± 33.84	43.34 ± 36.07^†^	33.52 ± 36.12	<0.001
Use of at least 1 long-acting antihypertensive drug administration in each time *N* (%)						<0.001
Only morning administration of short-acting drugs	57 (7.27)	8 (4.49)	8 (7.69)	13 (3.82)	86 (6.12)	
Only morning administration of long-acting drugs	348 (44.39)	51 (28.65)	31 (29.81)	85 (25.00)	515 (36.63)	
Only evening administration of short-acting drugs	26 (3.32)	13 (7.30)	2 (1.92)	13 (3.82)	54 (3.84)	
Only evening administration of long-acting drugs	77 (9.82)	19 (10.67)	11 (10.58)	52 (15.29)	159 (11.31)	
Both morning and evening administration of short-acting drugs	18 (2.30)	2 (1.12)	2 (1.92)	5 (1.47)	27 (1.92)	
Both morning and evening administration of long-acting drugs	258 (32.91)	85 (47.75)	50 (48.08)	172 (50.59)	565 (40.18)	

TIS, total antihypertensive therapeutic intensity score. ^*∗*^*p* value for comparing parameters of all home blood pressure phenotypes. ^†^*p* value <0.05 for comparing to controlled hypertension. ^‡^calculated by the following formula: TIS of all antihypertensive dr ugs taken in the evening(TIS(evening))/TIS of all antihypertensive dr ugs taken within 24 hours(TIS(24 hours).

**Table 3:** Blood pressure control according to office blood pressure target of <140/90 mmHg and average daytime home blood pressure target of <135/85 mmHg.

Number (%)	Office BP < 140/90 mmHg	Office BP ≥ 140/90 mmHg	Total
Home BP < 135/85 mmHg	495 (35.21)	422 (30.01)	917 (65.22)
Home BP ≥ 135/85 mmHg	137 (9.74)	352 (25.04)	489 (34.78)
Total	632 (44.95)	774 (55.05)	1,406 (100)

BP, blood pressure.

**Table 4:** Home blood pressure phenotypes and classical blood pressure phenotypes according to office blood pressure target of <140/90 mmHg and average daytime home blood pressure target of <135/85 mmHg.

Home BP phenotypes	Classical BP phenotypes				
	Well-controlled HT	WCHT	MHT	SHT	Total
Controlled HT	437 (88.28)	347 (82.23)	0 (0)	0 (0)	784 (55.76)
MoHT	36 (7.27)	42 (9.95)	24 (17.52)	76 (21.59)	178 (12.66)
EHT	22 (4.44)	33 (7.82)	18 (13.14)	31 (8.81)	104 (7.40)
MoEHT	0 (0)	0 (0)	95 (69.34)	245 (69.60)	340 (24.18)
Total	497 (100)	422 (100)	137 (100)	354 (100)	1,406 (100)

BP, blood pressure; HT, hypertension; WCHT, white-coat uncontrolled hypertension; MHT, masked uncontrolled hypertension; SHT, sustained uncontrolled hypertension; MoHT, Isolated uncontrolled morning hypertension; EHT, Isolated uncontrolled evening hypertension; MoEHT, combined morning-evening uncontrolled hypertension.

**Table 5:** Univariable analysis for the association of clinical factors and home blood pressure phenotypes (home blood pressure target of <135/85 mmHg).

Clinical factors	Isolated uncontrolled morning hypertension OR (95% CI)^*∗*^	*p* value^*∗*^	Isolated uncontrolled evening hypertension OR (95% CI)^*∗*^	*p* value^*∗*^	Combined morning-evening uncontrolled hypertension OR (95% CI)^*∗*^	*p* value^*∗*^
Age	1.00 (0.99–1.01)	0.860	1.00 (0.98–1.01)	0.806	0.98 (0.97–0.99)	<0.001
Male	1.30 (0.93–1.81)	0.121	1.20 (0.79–1.82)	0.389	1.25 (0.97–1.62)	0.090
Overweight	1.52 (1.04–2.21)	0.030	1.16 (0.74–1.83)	0.513	1.47 (1.10–1.96)	0.009
Diabetes mellitus	1.07 (0.73–1.58)	0.730	1.36 (0.86–2.16)	0.187	1.12 (0.83–1.51)	0.457
Dyslipidemia	1.15 (0.77–1.72)	0.486	1.37 (0.81–2.32)	0.237	1.20 (0.88–1.64)	0.242
Previous CVD^†^	1.53 (0.91–2.55)	0.105	1.01 (0.49–2.10)	0.974	1.19 (0.77–1.83)	0.438
Obstructive sleep apnea	1.30 (0.69–2.47)	0.418	1.55 (0.73–3.27)	0.252	1.81 (1.14–2.89)	0.012
Alcohol drinking	1.11 (0.45–2.75)	0.828	0.91 (0.27–3.09)	0.886	2.40 (1.34–4.29)	0.003
Numbers of antihypertensive classes	1.16 (1.00–1.35)	0.045	1.32 (1.10–1.57)	0.002	1.25 (1.11–1.40)	<0.001
Use of diuretics	1.42 (0.88–2.29)	0.155	1.94 (1.12–3.36)	0.018	1.32 (0.90–1.95)	0.157
Use of MRA	1.10 (0.41–2.98)	0.845	3.18 (1.36–7.43)	0.007	2.51 (1.34–4.70)	0.004
Use of RAAS inhibitors	1.00 (0.71–1.40)	0.993	1.18 (0.77–1.83)	0.450	0.95 (0.73–1.24)	0.709
Use of CCBs	1.01 (0.69–1.48)	0.971	1.39 (0.82–2.35)	0.216	1.42 (1.03–1.96)	0.032
Use of BBs	1.11 (0.78–1.58)	0.546	0.83 (0.52–1.32)	0.429	0.90 (0.68–1.20)	0.482
Use of peripheral alpha-I blockers	1.82 (1.19–2.77)	0.006	2.51 (1.54–4.09)	<0.001	2.54 (1.84–3.51)	<0.001
Use of other classes of antihypertensive medications	1.95 (0.97–3.92)	0.060	1.36 (0.51–3.61)	0.533	2.14 (1.23–3.73)	0.007
Timing of drug administration						
Only morning administration	Reference		Reference		Reference	
Only evening administration	2.13 (1.32–3.45)	0.002	1.31 (0.67–2.55)	0.424	2.61 (1.78–3.82)	<0.001
Both morning and evening administration	2.16 (1.50–3.11)	<0.001	1.96 (1.26–3.05)	0.003	2.65 (1.98–3.54)	<0.001
Evening to 24 hours dose ratio	2.83 (1.82–4.42)	<0.001	1.82 (1.03–3.21)	0.041	3.38 (2.37–4.80)	<0.001
Use of at least 1 long-acting antihypertensive drug administration each time						
Only morning administration of short-acting drugs	Reference		Reference		Reference	
Only morning administration of long-acting drugs	1.04 (0.47–2.32)	0.915	0.63 (0.28–1.45)	0.281	1.07 (0.56–2.05)	0.836
Only evening administration of short-acting drugs	3.56 (1.32–9.64)	0.012	0.55 (0.11–2.76)	0.466	2.19 (0.89–5.38)	0.087
Only evening administration of long-acting drugs	1.76 (0.72–4.30)	0.216	1.02 (0.38–2.69)	0.972	2.96 (1.47–5.95)	0.002
Both morning and evening administration of short-acting drugs	0.79 (0.15–4.07)	0.780	0.79 (0.15–4.07)	0.780	1.22 (0.38–3.88)	0.739
Both morning and evening administration of long-acting drugs	2.35 (1.08–5.12)	0.032	1.38 (0.62–3.07)	0.429	2.92 (1.55–5.50)	0.001
FBS	1.04 (0.92–1.18)	0.549	1.03 (0.88–1.21)	0.681	1.04 (0.94–1.15)	0.466
LDL-C	1.22 (1.01–1.47)	0.039	0.99 (0.77–1.26)	0.914	1.21 (1.04–1.40)	0.012
eGFR	0.99 (0.99–1.00)	0.126	0.99 (0.98–1.00)	0.057	0.99 (0.99–1.00)	0.013
Presence of albuminuria	1.16 (0.75–1.82)	0.504	1.70 (1.03–2.80)	0.039	2.22 (1.64–3.02)	<0.001

BMI, body mass index; CVD, cardiovascular disease; MRA, mineralocorticoid receptor antagonist; RAAS, renin-angiotensin-aldosterone system; CCBs, calcium channel blockers; BBs, beta-blockers; FBS, fasting blood sugar; LDL-C, low-density lipoprotein-cholesterol; eGFR, estimated glomerular filtration rate; SBP, systolic blood pressure; DBP, diastolic blood pressure. ^*∗*^compared with the controlled hypertensive group. ^†^included previous history of myocardial infarction, heart failure, ischemic stroke, or hemorrhagic stroke.

**Table 6:** Multivariable analysis for the association of clinical factors and home blood pressure phenotypes (home blood pressure target of <135/85 mmHg).

Clinical factors	Isolated uncontrolled morning hypertension adjusted OR (95% CI)^*∗*^	*p* value^*∗*^	Isolated uncontrolled evening hypertension adjusted OR (95% CI)^*∗*^	*p* value^*∗*^	Combined morning-evening uncontrolled hypertension adjusted OR (95% CI)^*∗*^	*p* value^*∗*^
Age	1.01 (0.98–1.04)	0.474	0.98 (0.95–1.02)	0.325	0.99 (0.95–1.02)	0.464
Male	1.00 (0.54–1.85)	0.987	0.86 (0.44–1.70)	0.668	0.65 (0.31–1.37)	0.255
Overweight	0.82 (0.43–1.57)	0.550	0.69 (0.33–1.42)	0.315	0.71 (0.32–1.57)	0.400
Diabetes mellitus	1.18 (0.49–2.82)	0.710	2.13 (0.86–5.29)	0.104	1.67 (0.59–4.76)	0.334
Dyslipidemia	0.76 (0.36–1.59)	0.463	1.17 (0.49–2.80)	0.718	0.62 (0.26–1.49)	0.286
Previous CVD^†^	5.54 (2.02–15.22)	0.001	0.87 (0.23–3.38)	0.845	2.39 (0.66–8.64)	0.183
Obstructive sleep apnea	0.71 (0.26–1.99)	0.517	1.45 (0.52–4.08)	0.478	1.34 (0.43–4.12)	0.613
Alcohol drinking	0.41 (0.10–1.65)	0.210	0.48 (0.10–2.28)	0.353	0.79 (0.19–3.24)	0.740
Numbers of antihypertensive classes	1.19 (0.26–5.43)	0.818	1.43 (0.30–6.78)	0.653	1.16 (0.19–7.23)	0.872
Use of diuretics	4.75 (0.83–27.30)	0.081	2.71 (0.45–16.23)	0.274	4.76 (0.58–39.07)	0.147
Use of MRA	0.19 (0.02–2.00)	0.166	1.04 (0.12–9.20)	0.972	0.80 (0.06–11.69)	0.873
Use of RAAS inhibitors	0.64 (0.12–3.36)	0.601	1.14 (0.20–6.50)	0.881	0.91 (0.12–6.57)	0.922
Use of CCBs	0.54 (0.10–2.94)	0.475	0.87 (0.14–5.21)	0.877	1.05 (0.13–8.24)	0.960
Use of BBs	0.55 (0.11–2.70)	0.463	0.31 (0.06–1.61)	0.164	0.31 (0.04–2.17)	0.236
Use of peripheral alpha-I blockers	0.35 (0.06–2.15)	0.258	1.31 (0.20–8.71)	0.778	0.83 (0.10–6.86)	0.866
Use of other classes of antihypertensive medications	2.63 (0.26–26.22)	0.409	1.14 (0.08–15.53)	0.923	1.54 (0.08–28.72)	0.774
Timing of drug administration						
Only morning administration	Reference		Reference		Reference	
Only evening administration	0.60 (0.03–13.72)	0.749	1.32 (0.04–39.18)	0.872	1.06 (0.03–43.10)	0.973
Both morning and evening administration	1.06 (0.21–5.45)	0.945	1.09 (0.19–6.31)	0.924	1.41 (0.20–9.98)	0.730
Use of at least 1 long-acting antihypertensive drug administration each time						
Only morning administration of short-acting drugs	Reference		Reference		Reference	
Only morning administration of long-acting drugs	0.95 (0.22–4.14)	0.942	0.20 (0.05–0.82)	0.025	0.20 (0.04–1.00)	0.049
Only evening administration of short-acting drugs	0.94 (0.03–28.90)	0.973	0.48 (0.01–21.96)	0.708	0.32 (0.01–19.18)	0.586
Only evening administration of long-acting drugs	0.31 (0.01–9.41)	0.500	0.30 (0.01–11.24)	0.515	0.23 (0.00–12.36)	0.471
Both morning and evening administration of short-acting drugs	0.13 (0.01–3.27)	0.216	0.29 (0.01–6.30)	0.434	0.15 (0.00–5.24)	0.299
Both morning and evening administration of long-acting drugs	1.03 (0.12–8.94)	0.982	0.25 (0.03–2.28)	0.220	0.33 (0.03–3.92)	0.383
Evening to 24 hours dose ratio	4.42 (0.20–96.56)	0.344	0.95 (0.03–26.81)	0.977	2.06 (0.05–78.38)	0.697
FBS	1.00 (0.98–1.01)	0.734	0.99 (0.97–1.01)	0.306	0.99 (0.97–1.01)	0.365
LDL-C	1.00 (0.99–1.01)	0.442	1.00 (0.99–1.01)	0.554	0.99 (0.98–1.00)	0.203
eGFR	1.00 (0.98–1.02)	0.937	1.00 (0.98–1.02)	0.871	1.00 (0.98–1.02)	0.828
Presence of albuminuria	1.13 (0.55–2.30)	0.737	1.30 (0.61–2.79)	0.499	1.59 (0.70–3.60)	0.269

BMI, body mass index; CVD, cardiovascular disease; MRA, mineralocorticoid receptor antagonist; RAAS, renin-angiotensin-aldosterone system; CCBs, calcium channel blockers; BBs, beta-blockers; FBS, fasting blood sugar; LDL-C, low-density lipoprotein-cholesterol; eGFR, estimated glomerular filtration rate; SBP, systolic blood pressure; DBP, diastolic blood pressure. ^*∗*^compared with the controlled hypertensive group and adjusted by both average office and daytime home BPs. †included previous history of myocardial infarction, heart failure, ischemic stroke, or hemorrhagic stroke.

## Data Availability

The datasets of this study are available for the only investigators of this study. The data are not publicly available. However, the authors consider providing these data upon reasonable request.

## Supplementary Materials

Supplementary Materials Supplementary Table 1: baseline characteristics of home blood pressure phenotypes by using a home blood pressure target of <130/80 mmHg. Supplementary Table 2: a regimen of prescribed antihypertensive drugs of home blood pressure phenotypes by using a home blood pressure target of <130/80 mmHg. Supplementary Table 3: blood pressure control according to office blood pressure and average daytime home blood pressure target of <130/80 mmHg. Supplementary Table 4: home blood pressure phenotypes and classical blood pressure phenotypes according to office and average daytime home blood pressure target of <130/80 mmHg. Supplementary Table 5: univariable analysis for the association of clinical factors and home blood pressure phenotypes (home blood pressure target of <130/80 mmHg). Supplementary Table 6: multivariable analysis for the association of clinical factors and home blood pressure phenotypes (home blood pressure target of <130/80 mmHg).
