# Supplementary material for: Prevalence and Clinical Characteristics including Patterns of Antihypertensive Drug Administration of the Different Home Blood Pressure Phenotypes in Treated Hypertensive Patients
Source: Int J Hypertens. 2022 Dec 8;2022:6912839. doi: 10.1155/2022/6912839 (PMC9754835; doi:10.1155/2022/6912839)
Supplement: Supplementary Materials — Supplementary Table 1: baseline characteristics of home blood pressure phenotypes by using a home blood pressure target of <130/80 mmHg. Supplementary Table 2: a regimen of prescribed antihypertensive drugs of home blood pressure phenotypes by using a home blood pressure target of <130/80 mmHg. Supplementary Table 3: blood pressure control according to office blood pressure and average daytime home blood pressure target of <130/80 mmHg. Supplementary Table 4: home blood pressure phenotypes and classical blood pressure phenotypes according to office and average daytime home blood pressure target of <130/80 mmHg. Supplementary Table 5: univariable analysis for the association of clinical factors and home blood pressure phenotypes (home blood pressure target of <130/80 mmHg). Supplementary Table 6: multivariable analysis for the association of clinical factors and home blood pressure phenotypes (home blood pressure target of <130/80 mmHg). [file 6912839.f1.docx]

**Supplementary Table** 1 **Baseline characteristics of home blood pressure phenotypes by using home blood pressure target of < 130/80 mmHg**

| **Parameters** | **Home blood pressure phenotypes** | | | | **Total** | **p-value^*^** |
| --- | --- | --- | --- | --- | --- | --- |
|  | **Controlled hypertension** | **Isolated uncontrolled morning hypertension** | **Isolated uncontrolled evening hypertension** | **Combined morning-evening uncontrolled hypertension** |  |  |
| Number (%) | 501 (35.63) | 201 (14.30) | 96 (6.83) | 608 (43.24) | 1,406 (100) | - |
| Age (years) | 64.33 + 13.03 | 65.52 + 11.86 | 64.18 + 14.35 | 60.74 + 14.98^†‡^ | 62.94 + 13.97 | < 0.001 |
| Male N (%) | 170 (33.93) | 75 (37.31) | 38 (39.58) | 271 (44.57)^†^ | 554 (39.40) | 0.004 |
| BMI (kg/m^2^) | 24.84 + 4.34 | 25.32 + 3.87 | 25.05 + 4.49 | 26.28 + 4.77^†^ | 25.54 + 4.52 | < 0.001 |
| Comorbidities N (%) | | | | | | |
| Overweight  (BMI > 23 kg/m^2^) | 308 (62.99) | 140 (71.43)^†^ | 58 (64.44) | 444 (75.38)^†^ | 950 (69.65) | < 0.001 |
| Diabetes mellitus | 114 (22.80) | 51 (25.37) | 24 (25.00) | 136 (22.44) | 325 (23.16) | 0.814 |
| Dyslipidemia | 388 (77.45) | 157 (78.11) | 72 (75.00) | 478 (78.88) | 1,095 (77.99) | 0.833 |
| Previous CVD** | 46 (9.20) | 19 (9.45) | 10 (10.42) | 57 (9.41) | 132 (9.41) | 0.987 |
| Chronic kidney disease | 73 (14.60) | 26 (12.94) | 24 (25.00)^†‡^ | 121 (19.97)^†‡^ | 244 (17.39) | 0.007 |
| Obstructive sleep apnea | 27 (5.42) | 13 (6.50) | 7 (7.29) | 54 (8.91) | 101 (7.21) | 0.161 |
| Current smoking N (%) | 7 (1.48) | 4 (2.15) | 1 (1.10) | 11 (1.89) | 23 (1.73) | 0.881 |
| Alcohol drinking N (%) | 14 (2.96) | 3 (1.61) | 5 (5.49) | 35 (6.00)^†‡^ | 57 (4.28) | 0.020 |
| Numbers of antihypertensive classes | 2.01 + 0.99 | 2.06 + 1.12 | 2.14 + 1.19^†^ | 2.25 + 1.14^†^ | 2.13 + 1.09 | 0.006 |
| Diuretics N (%) | 53 (10.58) | 26 (12.94) | 12 (12.50) | 79 (12.99) | 170 (12.09) | 0.638 |
| MRA N (%) | 10 (2.00) | 7 (3.48) | 4 (4.17) | 33 (5.43)^†^ | 54 (3.84) | 0.031 |
| ACEIs N (%) | 85 (16.97) | 26 (12.94) | 12 (12.50) | 87 (14.31) | 210 (14.94) | 0.408 |
| ARBs N (%) | 238 (47.50) | 96 (47.76) | 50 (52.08) | 298 (49.01) | 682 (48.51) | 0.849 |
| DHP-CCBs N (%) | 368 (73.45) | 142 (70.65) | 71 (73.96) | 465 (76.48) | 1,046 (74.40) | 0.374 |
| Non-DHP-CCBs N (%) | 23 (4.59) | 15 (7.46) | 8 (8.33) | 41 (6.74) | 87 (6.19) | 0.278 |
| BBs N (%) | 149 (29.74) | 61 (30.35) | 22 (22.92) | 179 (29.44) | 411 (29.23) | 0.563 |
| Peripheral alpha-I blockers N (%) | 53 (10.58) | 30 (14.93)^†^ | 20 (20.83)^†^ | 145 (23.85)^†‡^ | 248 (17.64) | < 0.001 |
| Centrally acting alpha-II agonists N (%) | 12 (2.40) | 2 (1.00) | 4 (4.17) | 18 (2.96) | 36 (2.56) | 0.332 |
| Direct vasodilators N (%) | 14 (2.79) | 8 (3.98) | 2 (2.08) | 22 (3.62) | 46 (3.27) | 0.720 |
| Laboratory result | | | | | | |
| FBS (mmol/l) | 5.99 + 1.23 | 6.08 + 1.24 | 6.05 + 1.22 | 6.01 + 1.36 | 6.02 + 1.29 | 0.849 |
| HbA1C (%) | 5.99 + 0.79 | 6.09 + 0.71 | 6.07 + 0.74 | 6.07 + 0.95 | 6.05 + 0.85 | 0.064 |
| Cholesterol (mmol/l) | 4.70 + 0.95 | 4.74 + 0.87 | 4.40 + 1.01 | 4.89 + 1.07^†⁋^ | 4.77 + 1.00 | < 0.001 |
| Triglyceride (mmol/l) | 1.27 + 0.61 | 1.35 + 0.69 | 1.37 + 0.70^†^ | 1.45 + 0.70^†^ | 1.37 + 0.67 | < 0.001 |
| HDL-C (mmol/l) | 1.57 + 0.42 | 1.49 + 0.39 | 1.48 + 0.42 | 1.47 + 0.51^†^ | 1.51 + 0.46 | < 0.001 |
| LDL-C (mmol/l) | 2.57 + 0.85 | 2.67 + 0.78 | 2.46 + 0.81 | 2.80 + 0.92^†⁋^ | 2.68 + 0.88 | < 0.001 |
| eGFR (ml/min/1.73 m^2^) | 80.25 + 21.29 | 78.83 + 20.67 | 76.62 + 24.09 | 77.97 + 25.67 | 78.82 + 23.40 | 0.561 |
| Presence of albuminuria N (%) | 83 (20.70) | 26 (17.69) | 24 (31.17)^†‡^ | 163 (32.80) ^†‡^ | 296 (26.38) | < 0.001 |
| Office BP | | | | | | |
| Average office SBP (mmHg) | 137.21 + 15.38 | 143.69 + 15.24^†^ | 140.66 + 15.56^†^ | 146.18 + 15.42^†⁋^ | 142.25 + 15.89 | < 0.001 |
| Average office DBP (mmHg) | 74.65 + 10.35 | 78.51 + 11.16^†^ | 76.55 + 10.27 | 82.37 + 12.51^†‡⁋^ | 78.67 + 11.94 | < 0.001 |
| Morning home BP | | | | | | |
| Average morning home SBP (mmHg) | 118.42 + 7.12 | 131.78 + 8.59 | 123.67 + 5.79 | 137.06 + 11.00 | 128.75 + 12.41 | < 0.001 |
| Average morning home DBP (mmHg) | 70.17 + 6.83 | 79.11 + 8.17 | 71.90 + 7.49 | 83.09 + 9.87 | 77.15 + 10.35 | < 0.001 |
| Evening home BP | | | | | | |
| Average evening home SBP (mmHg) | 117.40 + 7.31 | 121.95 + 6.78 | 133.00 + 6.70 | 136.45 + 10.51 | 127.35 + 12.40 | < 0.001 |
| Average evening home DBP (mmHg) | 68.26 + 7.09 | 71.58 + 7.70 | 76.15 + 8.35 | 81.61 + 10.77 | 75.04 + 10.86 | < 0.001 |
| Daytime home BP | | | | | | |
| Average daytime home SBP (mmHg) | 117.91 + 6.47 | 126.86 + 6.50^†^ | 128.33 + 5.18^†^ | 136.74 + 10.02^†‡⁋^ | 128.05 + 11.65 | < 0.001 |
| Average daytime home DBP (mmHg) | 69.21 + 6.69 | 75.34 + 7.31^†^ | 74.02 + 7.39^†^ | 82.35 + 9.72^†‡⁋^ | 76.10 + 10.11 | < 0.001 |

**Abbreviation:** kg/m^2^, kilogram/meter^2^; CVD, cardiovascular disease; MRA, mineralocorticoid receptor antagonist; ACEIs, angiotensin converting enzyme inhibitors; ARBs, angiotensin-II receptor blockers; DHP-CCBs, dihydropyridine calcium channel blockers; Non-DHP-CCBs, non-dihydropyridine calcium channel blockers; BBs, beta-blockers; FBS, fasting blood sugar; HbA1C, Hemoglobin A1C; HDL-C, high density lipoprotein-cholesterol; LDL-C, low density lipoprotein-cholesterol; eGFR, estimated glomerular filtration rate; mg/g, milligram of albuminuria/gram of urine creatinine; BP, blood pressure; SBP, systolic blood pressure; DBP, diastolic blood pressure; mmHg, millimeter of mercury

*p-value for comparing parameters of all home blood pressure phenotypes.

**included previous history of myocardial infarction, heart failure, ischemic stroke, or hemorrhagic stroke.

^†^p-value < 0.05 for comparing to controlled hypertension

^‡^p-value < 0.05 for comparing to isolated uncontrolled morning hypertension

^⁋^ p-value < 0.05 for comparing to isolated uncontrolled evening hypertension

**Supplementary Table 2 Regimen of prescribed antihypertensive drugs of home blood pressure phenotypes by using home blood pressure target of < 130/80 mmHg**

| **Parameters** | **Home blood pressure phenotypes** | | | | **Total** | **p-value^*^** |
| --- | --- | --- | --- | --- | --- | --- |
|  | **Controlled hypertension** | **Isolated uncontrolled morning hypertension** | **Isolated uncontrolled evening hypertension** | **Combined morning-evening uncontrolled hypertension** |  |  |
| Number (%) | 501 (35.63) | 201 (14.30) | 96 (6.83) | 608 (43.24) | 1,406 (100) | - |
| Frequency of drug administration (times per day) N (%) | | | | | | < 0.001 |
| 1 | 337 (67.27) | 114 (56.72) | 56 (58.33) | 303 (49.84) | 810 (57.61) |  |
| 2 | 142 (28.34) | 72 (35.82) | 31 (32.29) | 244 (40.13) | 489 (34.78) |  |
| 3 | 16 (3.19) | 10 (4.98) | 8 (8.33) | 41 (6.74) | 75 (5.33) |  |
| 4 | 6 (1.20) | 5 (2.49) | 1 (1.04) | 20 (3.29) | 32 (2.28) |  |
| Timing of antihypertensive drug administration N (%) | | | | | | < 0.001 |
| Only morning administration | 272 (54.29) | 94 (46.77) | 39 (40.63) | 196 (32.24) | 601 (42.75) |  |
| Only evening administration | 67 (13.37) | 20 (9.95) | 18 (18.75) | 108 (17.76) | 213 (15.15) |  |
| Both morning and evening administration | 162 (32.34) | 87 (43.28) | 39 (40.63) | 304 (50.00) | 592 (42.11) |  |
| Evening drug administration N(%) | 229 (45.71) | 107 (53.23) | 57 (59.38)^†^ | 412 (67.76)^†‡^ | 805 (57.25) | < 0.001 |
| Proportion of TIS (evening) to TIS (24 hours)^‡^ (%) | 26.18 + 35.36 | 29.42 + 33.40 | 37.75 + 38.42^†^ | 40.26 + 35.96^†‡^ | 33.52 + 36.12 | < 0.001 |
| Use of least 1 long-acting antihypertensive drug administration in each time N (%) | | | | | | < 0.001 |
| Only morning administration of short-acting drugs | 36 (7.19) | 16 (7.96) | 8 (8.33) | 26 (4.28) | 86 (6.12) |  |
| Only morning administration of long-acting drugs | 236 (47.11) | 78 (38.81) | 31 (32.29) | 170 (27.96) | 515 (36.63) |  |
| Only evening administration of short-acting drugs | 18 (3.59) | 6 (2.99) | 4 (4.17) | 26 (4.28) | 54 (3.84) |  |
| Only evening administration of long-acting drugs | 49 (9.78) | 14 (6.97) | 14 (14.58) | 82 (13.49) | 159 (11.31) |  |
| Both morning and evening administration of short-acting drugs | 9 (1.80) | 7 (3.48) | 1 (1.04) | 10 (1.64) | 27 (1.92) |  |
| Both morning and evening administration of long-acting drugs | 153 (30.54) | 80 (39.80) | 38 (39.58) | 294 (48.36) | 565 (40.18) |  |

**Abbreviation:** TIS, total antihypertensive therapeutic intensity score

*p-value for comparing parameters of all home blood pressure phenotypes

^†^p-value < 0.05 for comparing to controlled hypertension

^‡^calculated by the following formula: $\frac{TIS of all antihypertensive drugs taking in the evening (TIS (evening))}{TIS of all antihypertensive drugs taking within 24 hours (TIS (24 hours)}$

**Supplementary Table 3** **Blood pressure control according to office blood pressure and average daytime home blood pressure target of < 130/80 mmHg**

| **Number (%)** | **Office BP < 130/80 mmHg** | **Office BP > 130/80 mmHg** | **Total** |
| --- | --- | --- | --- |
| **Home BP < 130/80 mmHg** | 176 (12.52) | 462 (32.86) | 638 (45.38) |
| **Home BP > 130/80 mmHg** | 83 (5.90) | 685 (48.72) | 768 (54.62) |
| **Total** | 259 (18.42) | 1,147 (81.58) | 1,406 (100) |

**Abbreviation:** BP, blood pressure

**Supplementary Table 4 Home blood pressure phenotypes and classical blood pressure phenotypes according to office and average daytime home blood pressure target of < 130/80 mmHg**

| **Classical BP**  **phenotypes**  **Home BP**  **phenotypes** | **Well controlled HT** | **WCHT** | **MHT** | **SHT** | **Total** |
| --- | --- | --- | --- | --- | --- |
| **Controlled HT** | 147 (83.52) | 354 (76.62) | 0 (0) | 0 (0) | 501 (35.63) |
| **MoHT** | 19 (10.80) | 76 (16.45) | 13 (15.66) | 93 (13.58) | 201 (14.30) |
| **EHT** | 10 (5.68) | 32 (6.93) | 12 (14.46) | 42 (6.13) | 96 (6.83) |
| **MoEHT** | 0 (0) | 0 (0) | 58 (69.88) | 550 (80.29) | 608 (43.24) |
| **Total** | 176 (100) | 462 (100) | 137 (100) | 354 (100) | 1,406 (100) |

**Abbreviation:** BP, blood pressure; HT, hypertension; WCHT, white-coat uncontrolled hypertension; MHT, masked uncontrolled hypertension; SHT, sustained uncontrolled hypertension; MoHT, Isolated uncontrolled morning hypertension; EHT, Isolated uncontrolled evening hypertension; MoEHT, Combined morning-evening uncontrolled hypertension

**Supplementary Table 5 Univariable analysis for the association of clinical factors and home blood pressure phenotypes (home blood pressure target of < 130/80 mmHg)**

| **Clinical factors** | **Isolated uncontrolled morning hypertension**  **OR (95% CI)*** | **p-value*** | **Isolated uncontrolled evening hypertension**  **OR (95% CI)*** | **p-value*** | **Combined morning-evening uncontrolled hypertension OR (95% CI)*** | **p-value*** |
| --- | --- | --- | --- | --- | --- | --- |
| Age | 1.01  (0.99 – 1.02) | 0.288 | 1.00  (0.98 – 1.02) | 0.919 | 0.98  (0.97 – 0.99) | < 0.001 |
| Male | 1.16  (0.82 – 1.63) | 0.396 | 1.28  (0.81 – 2.00) | 0.288 | 1.57  (1.23 – 2.00) | < 0.001 |
| Overweight | 1.47  (1.02 – 2.11) | 0.036 | 1.07  (0.67 – 1.70) | 0.792 | 1.80  (1.38 – 2.34) | < 0.001 |
| Diabetes mellitus | 1.15  (0.79 – 1.68) | 0.468 | 1.13  (0.68 – 1.87) | 0.640 | 0.98  (0.74 – 1.30) | 0.887 |
| Dyslipidemia | 1.04  (0.70 – 1.54) | 0.849 | 0.87  (0.53 – 1.45) | 0.602 | 1.09  (0.82 – 1.45) | 0.565 |
| Previous CVD^†^ | 1.03  (0.59 – 1.81) | 0.917 | 1.15  (0.56 – 2.36) | 0.708 | 1.02  (0.68 – 1.54) | 0.907 |
| Obstructive sleep apnea | 1.21  (0.61 – 2.40) | 0.580 | 1.37  (0.58 – 3.25) | 0.472 | 1.71  (1.06 – 2.75) | 0.028 |
| Alcohol drinking | 0.54  (0.15 – 1.89) | 0.334 | 1.91  (0.67 – 5.43) | 0.227 | 2.09  (1.11 – 3.94) | 0.022 |
| Numbers of antihypertensive classes | 1.05  (0.90 – 1.23) | 0.534 | 1.12  (0.92 – 1.37) | 0.263 | 1.23  (1.10 – 1.38) | < 0.001 |
| Use of diuretics | 1.26  (0.76 – 2.07) | 0.373 | 1.21  (0.62 – 2.36) | 0.580 | 1.26  (0.87 – 1.83) | 0.217 |
| Use of MRA | 1.77  (0.66 – 4.72) | 0.253 | 2.13  (0.66 – 6.95) | 0.208 | 2.82  (1.38 – 5.78) | 0.005 |
| Use of RAAS inhibitors | 0.85  (0.61 – 1.19) | 0.348 | 1.00  (0.64 – 1.59) | 0.983 | 0.96  (0.75 – 1.23) | 0.734 |
| Use of CCBs | 0.86  (0.59 – 1.26) | 0.446 | 0.96  (0.57 – 1.60) | 0.864 | 1.26  (0.95 – 1.69) | 0.112 |
| Use of BBs | 1.03  (0.72 – 1.47) | 0.874 | 0.70  (0.42 – 1.17) | 0.177 | 0.99  (0.76 – 1.28) | 0.913 |
| Use of peripheral alpha-I blockers | 1.48  (0.92 – 2.40) | 0.109 | 2.22  (1.26 – 3.93) | 0.006 | 2.65  (1.88 – 3.72) | < 0.001 |
| Use of other classes of antihypertensive medications | 1.07  (0.48 – 2.38) | 0.866 | 0.99  (0.33 – 2.96) | 0.991 | 1.44  (0.83 – 2.50) | 0.196 |
| Timing of drug administration | | | | | | |
| Only morning administration | Reference |  | Reference |  | Reference |  |
| Only evening administration | 0.86  (0.50 – 1.50) | 0.603 | 1.87  (1.01 – 3.48) | 0.047 | 2.24  (1.57 – 3.19) | < 0.001 |
| Both morning and evening administration | 1.55  (1.09 – 2.21) | 0.014 | 1.68  (1.03 – 2.73) | 0.036 | 2.60  (2.00 – 3.39) | < 0.001 |
| Evening to 24 hours dose ratio | 1.33  (0.82 – 2.16) | 0.244 | 2.55  (1.40 – 4.66) | 0.002 | 3.05  (2.16 – 4.30) | < 0.001 |
| Use of at least 1 long-acting antihypertensive drug administration in each time | | | | | | |
| Only morning administration of short-acting drugs | Reference |  | Reference |  | Reference |  |
| Only morning administration of long-acting drugs | 0.74  (0.39 – 1.41) | 0.366 | 0.59  (0.25 – 1.39) | 0.227 | 1.00  (0.58 – 1.71) | 0.992 |
| Only evening administration of short-acting drugs | 0.75  (0.25 – 2.24) | 0.607 | 1.00  (0.27 – 3.77) | 1.000 | 2.00  (0.91 – 4.38) | 0.083 |
| Only evening administration of long-acting drugs | 0.64  (0.28 – 1.48) | 0.301 | 1.29  (0.49 – 3.39) | 0.611 | 2.32  (1.25 – 4.29) | 0.008 |
| Both morning and evening administration of short-acting drugs | 1.75  (0.55 – 5.53) | 0.340 | 0.50  (0.06 – 4.53) | 0.780 | 1.54  (0.55 – 4.32) | 0.413 |
| Both morning and evening administration of long-acting drugs | 1.18  (0.62 – 2.25) | 0.623 | 1.12  (0.48 – 2.60) | 0.796 | 2.66  (1.55 – 4.57) | < 0.001 |
| FBS | 1.06  (0.93 – 1.19) | 0.392 | 1.04  (0.88 – 1.23) | 0.649 | 1.02  (0.93 – 1.12) | 0.720 |
| LDL-C | 1.15  (0.95 – 1.40) | 0.151 | 0.85  (0.64 – 1.12) | 0.245 | 1.35  (1.17 – 1.56) | < 0.001 |
| eGFR | 1.00  (0.99 – 1.00) | 0.467 | 0.99  (0.98 – 1.00) | 0.166 | 1.00  (0.99 – 1.00) | 0.111 |
| Presence of albuminuria | 0.82  (0.51 – 1.34) | 0.434 | 1.73  (1.01 – 2.98) | 0.045 | 1.87  (1.38 – 2.54) | < 0.001 |

**Abbreviation:** BMI, body mass index; CVD, cardiovascular disease; MRA, mineralocorticoid receptor antagonist; RAAS, renin-angiotensin-aldosterone system; CCBs, calcium channel blockers; BBs, beta-blockers; FBS, fasting blood sugar; LDL-C, low density lipoprotein-cholesterol; eGFR, estimated glomerular filtration rate; SBP, systolic blood pressure; DBP, diastolic blood pressure

*compared with controlled hypertensive group.

^†^included previous history of myocardial infarction, heart failure, ischemic stroke, or hemorrhagic stroke.

**Supplementary Table 6 Multivariable analysis for the association of clinical factors and home blood pressure phenotypes (home blood pressure target of < 130/80 mmHg)**

| **Clinical factors** | **Isolated uncontrolled morning hypertension**  **Adjusted OR (95% CI)*** | **p-value*** | **Isolated uncontrolled evening hypertension**  **Adjusted OR (95% CI)*** | **p-value*** | **Combined morning-evening uncontrolled hypertension Adjusted OR (95% CI)*** | **p-value*** |
| --- | --- | --- | --- | --- | --- | --- |
| Age | 1.02  (0.99 – 1.06) | 0.135 | 0.98  (0.95 – 1.02) | 0.266 | 0.98  (0.94 – 1.01) | 0.162 |
| Male | 1.12  (0.61 – 2.04) | 0.721 | 1.17  (0.57 – 2.42) | 0.669 | 1.72  (0.86 – 3.43) | 0.125 |
| Overweight | 1.18  (0.65 – 2.12) | 0.588 | 0.83  (0.41 – 1.67) | 0.597 | 1.06  (0.53 – 2.12) | 0.870 |
| Diabetes mellitus | 1.55  (0.73 – 3.28) | 0.257 | 0.99  (0.38 – 2.60) | 0.989 | 1.47  (0.59 – 3.65) | 0.408 |
| Dyslipidemia | 0.71  (0.35 – 1.44) | 0.343 | 0.54  (0.23 – 1.27) | 0.158 | 0.72  (0.32 – 1.65) | 0.438 |
| Previous CVD^†^ | 2.41  (0.91 – 6.41) | 0.077 | 1.30  (0.38 – 4.45) | 0.680 | 1.40  (0.42 – 4.57) | 0.588 |
| Obstructive sleep apnea | 1.23  (0.45 – 3.41) | 0.685 | 1.05  (0.32 – 3.42) | 0.935 | 0.88  (0.29 – 2.73) | 0.826 |
| Alcohol drinking | 0.45  (0.08 – 2.44) | 0.353 | 1.27  (0.26 – 6.32) | 0.769 | 0.45  (0.09 – 2.36) | 0.347 |
| Numbers of antihypertensive classes | 1.97  (0.42 – 9.29) | 0.390 | 3.24  (0.63 – 16.56) | 0.157 | 2.90  (0.52 – 16.18) | 0.226 |
| Use of diuretics | 0.90  (0.14 – 5.70) | 0.909 | 2.28  (0.03 – 2.33) | 0.239 | 0.82  (0.11 – 6.29) | 0.849 |
| Use of MRA | 0.58  (0.06 – 6.10) | 0.651 | 0.25  (0.01 – 4.82) | 0.355 | 0.46  (0.04 – 5.87) | 0.548 |
| Use of RAAS inhibitors | 0.30  (0.06 – 1.66) | 0.169 | 0.48  (0.08 – 3.05) | 0.437 | 0.36  (0.05 – 2.45) | 0.298 |
| Use of CCBs | 0.28  (0.05 – 1.56) | 0.147 | 0.27  (0.04 – 1.75) | 0.171 | 0.36  (0.05 – 2.45) | 0.298 |
| Use of BBs | 0.40  (0.08 – 1.98) | 0.262 | 0.30  (0.06 – 1.57) | 0.152 | 0.26  (0.04 – 1.53) | 0.136 |
| Use of peripheral alpha-I blockers | 0.32  (0.05 – 2.15) | 0.241 | 0.25  (0.03 – 1.99) | 0.189 | 0.20  (0.02 – 1.65) | 0.134 |
| Use of other classes of antihypertensive medications | 0.32  (0.03 – 3.88) | 0.367 | 0.05  (0.00 – 1.38) | 0.076 | 0.32  (0.02 – 4.99) | 0.419 |
| Timing of drug administration | | | | | | |
| Only morning administration | Reference |  | Reference |  | Reference |  |
| Only evening administration | 0.90  (0.05 – 16.66) | 0.943 | 1.46  (0.04 – 60.17) | 0.843 | 5.87  (0.20 – 168.82) | 0.302 |
| Both morning and evening administration | 2.19  (0.48 – 9.99) | 0.312 | 1.13  (0.16 – 8.05) | 0.906 | 5.71  (0.99 – 32.99) | 0.051 |
| Use of at least 1 long-acting antihypertensive drug administration in each time | | | | | | |
| Only morning administration of short-acting drugs | Reference |  | Reference |  | Reference |  |
| Only morning administration of long-acting drugs | 0.38  (0.11 – 1.27) | 0.116 | 0.17  (0.04 – 0.69) | 0.013 | 0.23  (0.05 – 1.00) | 0.050 |
| Only evening administration of short-acting drugs | 0.58  (0.02 – 15.35) | 0.743 | 0.42  (0.01 – 24.21) | 0.677 | 1.86  (0.04 – 81.83) | 0.748 |
| Only evening administration of long-acting drugs | 0.34  (0.01 – 8.23) | 0.509 | 0.30  (0.01 – 15.22) | 0.515 | 1.60  (0.04 – 64.47) | 0.803 |
| Both morning and evening administration of short-acting drugs | 1.26  (0.12 – 13.07) | 0.849 | N/A^‡^ | N/A^‡^ | 1.72  (0.10 – 29.58) | 0.710 |
| Both morning and evening administration of long-acting drugs | 0.82  (0.12 – 5.75) | 0.838 | 0.25  (0.02 – 2.69) | 0.252 | 1.44  (0.15 – 14.24) | 0.757 |
| Evening to 24 hours dose ratio | 0.90  (0.05 – 15.37) | 0.943 | 1.33  (0.03 – 51.27) | 0.878 | 0.26  (0.01 – 7.12) | 0.428 |
| FBS | 0.99  (0.97 – 1.01) | 0.202 | 0.99  (0.97 – 1.01) | 0.267 | 0.98  (0.97 – 1.00) | 0.067 |
| LDL-C | 1.00  (0.99 – 1.01) | 0.624 | 1.00  (0.98 – 1.00) | 0.159 | 1.00  (1.00 – 1.01) | 0.315 |
| eGFR | 1.00  (0.98 – 1.02) | 0.967 | 0.99  (0.97 – 1.01) | 0.231 | 1.00  (0.98 – 1.02) | 0.975 |
| Presence of albuminuria | 0.84  (0.40 – 1.73) | 0.627 | 2.01  (0.88 – 4.60) | 0.097 | 1.87  (0.85 – 4.12) | 0.119 |

**Abbreviation:** BMI, body mass index; CVD, cardiovascular disease; MRA, mineralocorticoid receptor antagonist; RAAS, renin-angiotensin-aldosterone system; CCBs, calcium channel blockers; BBs, beta-blockers; FBS, fasting blood sugar; LDL-C, low density lipoprotein-cholesterol; eGFR, estimated glomerular filtration rate; SBP, systolic blood pressure; DBP, diastolic blood pressure

*compared with controlled hypertensive group and adjusted by both average office and daytime home BPs.

^†^included previous history of myocardial infarction, heart failure, ischemic stroke, or hemorrhagic stroke.

^‡^N/A, not applicable duo to too small sample size for calculation in multivariable regression
